# Reliability and validity of the Student Perceptions of School Cohesion Scale in a sample of Salvadoran secondary school students

**DOI:** 10.1186/1472-698X-9-30

**Published:** 2009-11-25

**Authors:** Andrew E Springer, Amy McQueen, Guillermo Quintanilla, Marcela Arrivillaga, Michael W Ross

**Affiliations:** 1 Michael & Susan Dell Center for Advancement of Healthy Living, University of Texas School of Public Health, Austin Regional Campus, Austin, Texas, USA; 2 Division of Health Behavior Research, Washington University, School of Medicine, St. Louis, Missouri, USA; 3 Departamento de Educación para la Salud, Universidad de El Salvador, San Salvador, El Salvador; 4 Departamento de Ciencias Sociales. Pontificia Universidad Javeriana Cali, Cali, Colombia; 5 Center for Health Promotion and Prevention Research, University of Texas School fo public Health, Houston Campus, Houston, Texas, USA

## Abstract

**Background:**

Despite a growing body of research from the United States and other industrialized countries on the inverse association between supportive social relationships in the school and youth risk behavior engagement, research on the measurement of supportive school social relationships in Central America is limited. We examined the psychometric properties of the *Student Perceptions of School Cohesion *(SPSC) scale, a 10-item scale that asks students to rate with a 5-point Likert-type response scale their perceptions of the school social environment, in a sample of public secondary school students (mean age = 15 years) living in central El Salvador.

**Methods:**

Students (n = 982) completed a self-administered questionnaire that included the SPSC scale along with measures of youth health risk behaviors based on the Center for Disease Control and Prevention's Youth Risk Behavior Survey. Exploratory factor analysis was used to assess the factor structure of the scale, and two internal consistency estimates of reliability were computed. Construct validity was assessed by examining whether students who reported low school cohesion were significantly more likely to report physical fighting and illicit drug use.

**Results:**

Results indicated that the SPSC scale has three latent factors, which explained 61.6% of the variance: *supportive school relationships, student-school connectedness, and student-teacher connectedness*. The full scale and three subscales had good internal consistency (r_s _= .87 and α = .84 for the full scale; r_s _and α between .71 and .75 for the three subscales). Significant associations were found between the full scale and all three subscales with physical fighting (p ≤ .001) and illicit drug use (p < .05).

**Conclusion:**

Findings provide evidence of reliability and validity of the SPSC for the measurement of supportive school relationships in Latino adolescents living in El Salvador. These findings provide a foundation for further research on school cohesion and health risk behavior in Latino adolescents living in the U.S. and other Latin American countries.

## Background

Youth risk behavior is an important indicator of the health of young people based on its association with several mortality and morbidity outcomes, including intentional injury stemming from aggression and suicidal ideation, chronic disease resulting from substance use and misuse, sexually transmitted disease, and undesired social outcomes such as unintended teenage pregnancy [1]. While the social sciences may be far from identifying an immunization against harmful behaviors connected to poor health outcomes, a growing body of literature in public health points to the importance of the social context for its potential to confer a protective effect against adverse health behaviors and health status. Supportive interpersonal relationships within the school context may hold specific relevance for protecting against a range of health risk behaviors among adolescents.

Psychosocial adolescent behavior theories such as social control [2] and social bonding theory [3], the Social Development Model [4], primary socialization theory [5], and resilience theory [6,7] highlight the important role of cohesive and supportive interpersonal relationships within an adolescent's primary socialization contexts, including the school context, for protecting against risk-taking behaviors. According to these theoretical perspectives, the quality of affective relationships--and specifically caring and supportive relationships--that one has with others contributes to the social bonds that subsequently reduce engagement in risk-taking behavior.

Empirical research from industrialized countries has indicated a protective effect of a supportive school environment and a student's connection to school on engagement in various youth risk behaviors. Social bonding of students, student school-connectedness, and a caring and supportive school climate have been found to be inversely associated with substance use and delinquency [8-12], aggression [10,11,13], sexual risk behavior [9,13], and depressive symptoms and suicidality [10,12,13]. In a four-year randomized controlled trial with 25 secondary schools in Australia, a school-based intervention designed specifically to promote social inclusion and commitment to education was found to significantly reduce risk behavior in adolescents, including substance use and early initiation of sexual intercourse [14].

Although past theoretical and empirical research provide strong evidence for the association between supportive school social climate and student-school connectedness with youth risk behaviors, several gaps in the literature remain. For example, some studies conceptualize supportive school relationships at the individual level in terms of a student's direct connection to school and the people within school [10], while other studies conceptualize social relationships at an ecological or contextual level in terms of a general school climate of caring and supportive relationships [8,15,16]. Measures that capture both individual-level connection to school as well as a contextual level of supportive climate are needed. On a related note, some research has found that different dimensions of school connectedness, such as connectedness with teachers vs. general connectedness with school, have different associations with the initiation of youth risk behavior such as substance use [13]. The development of measures that distinguish between levels and dimensions of social relationships within the school context may contribute to a better understanding of direct and indirect social influences on health behavior. Lastly, published research on the validity and reliability of instruments purporting to measure supportive school relationships with Central American adolescent populations is limited. Because Central American adolescents share many of the same risk behaviors as U.S. adolescents, such as drug use [17] and aggressive behavior such as physical fighting [18,19], research on the protective effects of specific social contexts such as schools holds the potential to guide intervention efforts for reducing youth risk behavior initiation in Central American youth.

To assess the associations between supportive school relationships and youth risk behaviors among Central American youth, it is important to construct valid and internally consistent measures of supportive school relationships that encompass both individual-level and contextual-level items, and to evaluate the measure with the intended target population. In contributing to the measurement of supportive school relationships in Central America, the current study examined the psychometric properties of the *Student Perceptions of School Cohesion *scale with a sample of public secondary school students living in the central region of El Salvador. The specific aims of the current study were to: 1) explore the factorial structure of the Student Perceptions of School Cohesion scale and determine if one or more latent factors characterized the set of scale items; 2) examine the internal consistency reliability of the scale(s); and 3) assess evidence of construct validity of the scale(s) by exploring the association between supportive school relationships and selected youth risk behavior outcomes.

## Method

### Instrument

The study is based on survey data collected as part of the *Salud y Bienestar de los Jóvenes en El Salvador *study [Health and Wellbeing of Youth in El Salvador]. The *Salud y Bienestar de los Jóvenes *self-administered questionnaire consisted of 66 closed-ended items with dichotomous and ordinal-level response choices to assess perceptions of school social environment and health risk behaviors among Salvadoran secondary school students. Ten items of the *Salud y Bienestar de los Jóvenes *questionnaire specifically assessed student perceptions of school cohesion and student-school connectedness. The current study focuses on these ten items with the aim of evaluating their psychometric scale properties. The ten *Student Perceptions of School Cohesion *[SPSC] items asked students to rate with a 5-point Likert-type response scale how strongly they agree or disagree with statements such as: "People care about each other in this school," "Students in my class work together to solve problems," and "Students and teachers at this school are close with each other" (see Table 1 for a full list of items). In the *Salud y Bienestar de los Jóvenes *questionnaire, the ten items were formatted under two consecutive questions, with the first six items in Table 1 grouped under one question and the remaining four items grouped under a separate question. The ten items were based in part on items from Battistich's and Hom's [8] "Students' sense of the school as a community" subscale of caring and supportive interpersonal relationships as well as items measuring student-school connectedness from the National Longitudinal Study on Adolescent Health [10].

**Table 1:** Item-total correlation and response distribution of Student Perceptions of School Cohesion scale among Salvadoran public secondary school students (n = 935)

How much do you agree or disagree with the following statements?	Item-Total Correlation	Strongly Agree	Somewhat Agree	Neutral^1^	Somewhat Disagree	Strongly Disagree
*¿'Qué tan de acuerdo o desacuerdo está usted con* *las siguientes afirmaciones?*		(%)	(%)	(%)	(%)	(%)
People care about each other in this school.	0.52	40.0	32.0	13.5	7.8	6.7
*La gente se preocupa uno por el otro en esta escuela.*						
Students support each other in this school.	0.55	47.5	34.7	7.0	6.7	4.1
*Los estudiantes se apoyan en esta escuela.*						
Teachers at this school are close to the students.	0.55	69.6	17.6	5.0	4.6	3.2
*Los maestros de esta escuela se identifican con los estudiantes.*						
Students feel very close to the teachers at this school.	0.55	53.6	30.0	7.5	6.1	2.8
*Los estudiantes se identifican mucho con los maestros de esta escuela.*						
Students in my class work together to solve problems.	0.48	52.2	28.8	7.2	5.7	6.2
*Los estudiantes en mi clase se ayudan en resolver problemas.*						
People in this school are willing to help each other.	0.61	42.3	33.9	10.7	7.9	5.3
*La gente en esta escuela tiene la voluntad de ayudarse*						
I can count on people in this school when I have a problem.	0.55	66.4	17.7	3.8	5.9	6.2
*Yo puedo contar con los compañeros y maestros en esta escuela.*						
I feel close to people at school.	0.56	59.2	27.1	5.8	3.9	4.0
*Yo me siento identificado(a) con los compañeros en esta escuela.*						
I feel a part of this school.	0.47	80.4	11.4	3.3	2.1	2.7
*Yo me siento parte de mi escuela*						
My teachers care about the work I do at school.	0.53	74.8	15.3	3.6	2.9	3.4
*Mis maestros se preocupan por el trabajo que hago.*						

^1^Neither agree nor disagree

Translation of response options: *Estoy muy de acuerdo; Estoy algo de acuerdo; Neutral: ni de acuerdo o en desacuerdo; No estoy muy de acuerdo; No estoy nada de acuerdo.*

All items measuring youth risk behaviors were selected from the instrument used for the U.S. Youth Risk Behavior Survey [20], a comprehensive adolescent health survey that has been administered biennially in the United States since 1991 by the Centers for Disease Control and Prevention. The current study examined two risk behaviors in relation to the SPSC scales to assess construct validity: aggressive behavior (physical fighting in the past 12 months) and illicit drug use (ever having consumed cocaine, marijuana, or inhalants). These risk behaviors were selected based on their associations in previous studies with student-school connectedness and supportive school climate [8-12,21]. Risk behaviors were coded as present/absent. In assessing the associations between the SPSC and risk behaviors, we took into account the following student demographic characteristics: gender, age, geographic location (urban/rural), and subjective economic status. Gender, age, and subjective economic status were determined based on self-reported measures; geographic location was determined according to school classifications from the Ministry of Education of El Salvador. Subjective economic status was adapted from a measure used in previous research with high school students [22] that asks students to rate their family's standard of living on a 5-point response scale, with 1= living very well off to 5 = poor.

All items were translated from English to Spanish and back-translated and centered with the assistance of two Salvadoran university students and a native English speaker fluent in Spanish. Content and face validity were assessed through discussions with national directors from the Ministry of Education of El Salvador and school principals to evaluate the appropriateness and relevance of the items within the Salvadoran school context. In order to assess the level of comprehension of the items, the questionnaire was pilot-tested with a separate sample of 35 urban eighth grade students attending a school in the study area. This pilot sample was not included as part of the main study sample.

### Sample and Data Collection

A multistage sampling frame was employed in which school districts, schools, and classrooms were randomly selected. Participants included eighth and ninth grade secondary school students attending 16 public schools in the central region of El Salvador who were present on the day of the study. The questionnaire was administered to students between June and August of 1999 according to standardized data collection procedures by a team comprising a technician from the National Training Center of the Ministry of Education of El Salvador, a Salvadoran university student, and the first author.

Student informed assent and parental informed consent were obtained for all participants prior to participation in the study, and collection of information was conducted in a confidential manner. Student assent was obtained by providing a verbal and written description of the study to all potential participants, which detailed the subject matter and placed specific emphasis on the study's voluntary and confidential nature. Under the informed assent procedure, students were informed that if they did not participate in the study, neither their grades in school nor their standing in school would be affected. Furthermore, students were informed that they did not have to answer any question they did not want to answer, that they could stop participating in the survey at any time, and that they would be provided an alternative activity if they did not want to participate in the survey. In addition to obtaining student assent for participation in the study, passive parental informed consent for participation in the study was obtained for all participants. At the time of the research, passive parental informed consent was an accepted protocol of the United States Centers for Disease Control and Prevention (CDC) in Atlanta, Georgia, for conducting research with adolescents in their *Youth Risk Behavior Survey *(YRBS), a national monitoring tool for assessing health risk behavior trends [20]. This consent procedure consists of sending a letter home with the student that describes the study and asks parents or guardians to submit a signed form only if they do not wish their child to participate in the study. In ensuring confidentiality of the participants, the survey was administered by trained data collectors who followed protocols that included separation of desks during the completion of the surveys as well as close monitoring of students during the completion of the survey. Names of participants and schools were not included on questionnaires nor connected in any way with questionnaire responses. The study protocol, along with the study objectives, methods, and English language version of the questionnaire, were reviewed and approved by the Committee for the Protection of Human Subjects of the University of Texas Health Science Center at Houston; the Spanish translation of the study objectives, methods and questionnaire were reviewed and approved by a team composed of Salvadoran parents and representatives of the Ministry of Education of El Salvador that was formed specifically to ensure the ethical conduct of the study as well as the protection of study participants.

### Data Analysis

To assess the dimensionality of the *Student Perceptions of School Cohesion *scale, an exploratory factor analysis was conducted. We used principal axis analysis in SPSS version 15.0 (Chicago, Il.) as the extraction method. Based on the relatively high correlations among the majority of items (> .3), an oblique rotation was performed using Promax. Three criteria from Green and Salkind [23] were applied to determine the number of factors to be retained: 1) the absolute values of the eigenvalues; 2) the relative values of the eigenvalues; and 3) the relative interpretability of the rotated solutions. In addition, a scree plot and the variance explained by the factor solution also were considered in making decisions to retain or exclude factors.

Two internal consistency estimates of reliability were computed for the Student Perceptions of School Cohesion scale: a split-half coefficient expressed as a Spearman-Brown corrected correlation and Cronbach's α. For the split-half coefficient, the scale was split into two halves by odd and even numbering of items with the goal of creating equivalent halves. The following criteria were used to evaluate coefficient α: α > 0.60 reflects modest internal consistency and α > 0.70 reflects good internal consistency for research purposes [24].

This study also assessed the construct validity of the scale(s). Construct validity is concerned with the theoretical relationship of a variable with other variables [25]. Adolescent psychosocial theories such as social control and bonding theories [2,3] and the Social Development Model [4] posit that weak bonds of attachment with conventional forms of society such as the school result in increased health risk and delinquency behaviors. In addition, an emerging body of empirical research has suggested that supportive school environment and social bonding among students at school are inversely related to several youth risk behaviors such as aggressive behavior and substance use [8-10].

Based on this theoretical and empirical foundation, and with the aim of assessing construct validity of SPSC scale(s), the SPSC scale scores were examined for their association with participation in physical fights in the past 12 months and lifetime illicit drug use. A composite variable was created for each of the subscales that was dichotomized according to strength of agreement with school cohesion statements, with high school cohesion defined as those who responded 'strongly agree' and 'somewhat agree' and low school cohesion defined as 'neutral', 'somewhat disagree', and 'strongly disagree'. Multiple logistic regression analyses were conducted to assess whether students who reported high school cohesion were significantly less likely to report physical fighting and illicit drug use than students who reported low school cohesion, adjusting for gender, age, urban/rural geographic location, and subjective economic status. Differences were considered statistically significant if the p value was < .05.

## Results

Among 1007 public school students present on the day of the questionnaire administration, 17 opted not to participate in the study and 8 students provided invalid or inconsistent responses (e.g., 'never smoked' and then responded 'smoked 20 or more times'), resulting in a total sample size of 982 completed questionnaires for analysis. The mean age of the respondents was 15 years (SD ± 1.4), and the sample had a slightly higher proportion of male students (52.6%).

### Descriptive Statistics

Item-by-item descriptive analyses of the SPSC scale indicate that responses were skewed toward more favorable perceptions of school cohesion (Table 1). Between 72% and 92% of the sample indicated a response of 'strongly agree' or 'somewhat agree' with the school cohesion items, with "people care about each other in this school" receiving the lowest favorable rating and "I feel a part of this school" receiving the highest rating.

### Exploratory Factor Analysis

Although "Kaiser's rule" suggests that only components with eigenvalues greater than one should be retained [26], this criterion may be too restrictive and may fail to identify potential factors. Results of this study indicated that eigenvalues for two of the ten components were greater than 1 (4.15 and 1.08), and one of the components approached 1 (.928). These three components accounted for a total of 61.6% of the variance, of which the third component explained 9%. The scree plot also suggested the presence of three separate factors before tapering off, indicating that the initial hypothesis of unidimensionality was incorrect. Based on the size of the eigenvalues, the variance explained, and the scree plot results, three factors were retained and rotated using Promax rotation. The correlations (factor loadings) of items in the final three factor model are reported in Table 2. These factors are interpretable from both a conceptual and theoretical basis. Factor 1, *supportive school relationships*, includes items that reflect supportive relationships at an ecological or contextual level, which may not reflect an individual's direct connection to that supportive context (Table 1). Subscale items include both peer and general social relationships at school. Factor 2, *student-school connectedness*, includes items that are representative of a student's direct connection to the overall school and to the people within the school. Lastly, Factor 3, *student-teacher connectedness*, represents emotional closeness between students and teachers at an ecological or contextual level.

**Table 2:** Correlations between the School Cohesion items and School Cohesion factors in a secondary school from central El Salvador (n = 935)

	Factors		
	
	Supportive School Relationships	Student-School Connectedness	Student-Teacher Connectedness
Students support each other in this school	**0.63**	0.05	-0.01
Students in my class work together to solve problems	**0.64**	-0.02	-0.04
People in this school are willing to help each other	**0.72**	-0.03	0.06
People care about each other in this school	**0.55**	0.07	0.01
I feel close to people at school	0.16	**0.47**	0.07
I feel a part of this school	-0.04	**0.47**	0.26
I can count on people in this school when I have a problem	0.12	**0.59**	-0.01
My teacher cares about the work I do at school	-0.06	**0.76**	-0.09
Teachers at this school are close to the students	-0.10	0.03	**0.87**
Students feel close to the teachers at this school	0.17	-0.08	**0.65**

*Based on Promax procedure for oblique rotation: pattern matrix

### Internal Consistency Reliability

The two internal consistency estimates of reliability computed for the Student Perception of School Cohesion scale provided similar results. For the "Supportive School Relationships" subscale, the values computed from the Spearman-Brown corrected correlation and coefficient alpha were .71 and .74, respectively. For the "Student-School Connectedness" subscale, the Spearman-Brown corrected correlation and coefficient alpha were .75 and .72, respectively. Lastly, for the "Student-Teacher Connectedness" subscale, the coefficient alpha was .72, which is equivalent to the Guttman-Flanagan split half; the Spearman-Brown coefficient was not calculated based on the presence of only two items. For the full scale, Spearman-Brown corrected correlation and coefficient alpha were .87 and .84, respectively. These values indicate good internal consistency.

### Construct Validity

Composite variables were created for each subscale identified in the exploratory factor analysis to assess potential evidence of construct validity of the SPSC subscales as per their associations with two adolescent risk behavior variables. The supportive school relationships and student-school connectedness composite variables ranged from 4 to 20 and were dichotomized according to strength of agreement, with 4 to 8 (high support/connectedness) equal to 'strongly agree' or 'somewhat agree' and 9 to 20 (low support/connectedness) equal to 'neutral', 'somewhat disagree', and 'strongly disagree'. The student-teacher connectedness variable ranged from 2 to 10, with high connectedness equal to 2 to 4 and low connectedness equal to 5 to 10.

Table 3 presents the associations of perceived school social cohesion with physical fighting and illicit drug use. In exploring the association between the full scale and the risk behavior outcomes, students with high perceptions of school social cohesion were significantly less likely to report both physical fighting in the last 12 months (Adjusted Odds Ratio (AOR) = 0.50, 95% Confidence Interval (CI): 0.35, 0.73, p < .001) and lifetime illicit drug use (AOR = 0.34, 95% CI: 0.20, 0.57, p < .001). We found the three supportive social relationship subscales had a similar protective effect on physical fighting. Student who reported high perceptions of supportive school relationships, student-school connectedness, and student-teacher connectedness were significantly less likely to report engagement in a physical fight in the past 12 months (AOR = 0.56, 95% CI: 0.41, 0.78; AOR = 0.49, 95% CI: 0.33, 0.75; and AOR = 0.54, 95% CI: 0.37, 0.79, respectively, p ≤ .001). In assessing the associations between the composite variables and illicit drug use, students with high perceptions of supportive school relationships reported significantly less illicit drug use (OR = 0.48, 95% CI: 0.29, 0.77, p = .001). Less robust but still significant inverse associations were found for illicit drug use with student-school connectedness (OR = 0.47, 95% CI: 0.26, 0.84, p = .01) and student-teacher connectedness (OR = 0.55, 95% CI: 0.32, 0.95, p = .03) (Table 3).

**Table 3:** Percentage of public secondary school students from central El Salvador who reported engaging in a physical fight and illicit drug use by level of perceived social cohesion within school and student-school connectedness. (n = 935)

	Engaged in a physical fight in past 12 months		Ever consumed illicit drugs	
	%	AOR (95% C.I.)^c^	%	AOR (95% C.I.)^c^
*Full scale*				
School social cohesion				
High Perceptions^a^	27.5***	0.50 (0.35, 0.73)	6.5***	0.34 (0.20, 0.57)
Low Perceptions^b ^(referent)	42.5***	1.00	15.4***	1.00
*Sub-scales*				
Supportive school relationships				
High Perceptions^a^	28.2***	0.56 (0.41, 0.78)	6.9***	0.48 (0.29, 0.77)
Low Perceptions^b ^(referent)	39.6***	1.00	12.0***	1.00
Student-school connectedness				
High Perceptions^a^	29.0***	0.49 (0.33, 0.75)	7.8*	0.47 (0.26, 0.84)
Low Perceptions^b ^(referent)	41.8***	1.00	12.7*	1.00
Student-teacher connectedness				
High Perceptions	29.7***	0.54 (0.37, 0.79)	7.9*	0.55 (0.32, 0.95)
Low Perceptions^b ^(referent)	44.0***	1.00	12.7*	1.00

Bold font indicates statistical significance at p < .05.

*p < .05; **p < .01, ***p ≤ .001 based on Logistic Regression Analyses.

^a^Represents students who responded "Strongly agree" or "somewhat agree" based on five-point scale

^b^Represents students who responded "Strongly disagree", "somewhat disagree" or "neutral" based on 5-point scale.^c^Adjusted Odds Ratio. Adjusted for gender, age, rural/urban status, and perceived socio-economic status.

## Discussion

Although several efforts have been made to develop instruments to measure quality of life and well-being of students within schools [8,27-29], few studies have examined well-being as related to school social relationships in a Central American school context. This study explored the psychometric properties of the Student Perceptions of School Cohesion [SPSC] scale -a scale that focuses specifically on student connectedness and caring and supportive social relationships within the school context--based on a sample of public secondary school students living in the central region of El Salvador. Findings from this study indicated that the SPSC scale has three latent factors related to *supportive school relationships, student-school connectedness*, and *student-teacher connectedness *and that these subscales as well as the full SPSC scale have good internal consistency. The construct validity of the SPSC scale is further strengthened by the observed associations between school social environment and youth risk behaviors found with the full scale and all three subscales with physical fighting and illicit drug use.

The three factors identified in the rotated solution, supportive school relationships, student-school connectedness, and student-teacher connectedness, are interpretable from both a conceptual and theoretical basis. Items for the first factor stem from Battistich's and Hom's [8] "Students' sense of the school as a community" subscale of caring and supportive interpersonal relationships, which provides a more ecological measure of school climate. In addition to empirical findings of the importance of supportive school social relationships at an ecological level for reducing risk behaviors such as drug use and delinquency [see [8,9]], this contextual level of social cohesion, with or without direct individual-to-school ties, may provide a sense of social belongingness for adolescents. Perceptions of social belongingness, even in the absence of direct interpersonal social ties, have been cited by sociological theorists as important for psychological well-being [30].

Our findings on the remaining SPSC scale items, which stem from research on school connectedness [10], indicate that these items represent two different latent factors: student-school connectedness and student-teacher connectedness. A student's level of connectedness to school has been found to be protective against a range of youth risk behaviors [10-12,21]. The distinction of student-school connectedness and student-teacher connectedness may be important given findings by McNeely and Falci [13] that indicated a protective effect of teacher support on adolescent risk behavior but no effect of student connectedness to school. Our research also indicates that supportive social relationships at school represents a multidimensional construct that may differ in terms of school relationships at an ecological or contextual level, a student's direct connection to the school and people within the school, and a student's connection to his/her teacher.

Findings of the current study provide some evidence of construct validity for the use of the Student Perceptions of School Cohesion scale in measuring supportive school social relationships. Our findings of an inverse relation between the subscale of supportive school relationships and aggression mirror findings from Battistich and Hom [8] in their study of fifth and sixth graders in the United States. We also found inverse associations between student-school connectedness and student-teacher connectedness and aggression- findings supported by previous research [10,13,31]. The observed associations between the full SPSC scale and the supportive school relationships subscale and illicit drug use are also similar to findings by McBride et al. [9] regarding higher student social bonding at school and lower illicit drug use.

Several possible explanations may account for the less robust but still significant associations found between the student-school connectedness and student-teacher connectedness with illicit drug use. First, the illicit drug use measure was based on lifetime drug use; therefore, this measure may not have been specific enough to capture the influence of school connectedness on this behavior. Distinguishing between heavy drug users and experimental drug users might provide more information about the influence of school relationships on illicit drug use. Second, it is possible that the different levels and forms of social relationships at school may affect drug use differently. Feeling connected or not connected to classmates and teachers may be less important for drug use than perceiving supportive relationships within the broader school context, as suggested in this study. As such, students who perceive low support in the school environment, despite their individual connection to school, may seek out relationships outside of school to fill a social connectedness void. Lightfoot's [[32], p.10] qualitative research on adolescents and risk taking suggests that adolescents engage in risk behaviors with peers as a way of promoting cohesion, trust, and closeness in initiating new relationships and consolidating existing relationships. Engaging in substance use with peers may be one approach for developing connectedness with other adolescents when social connectedness is not perceived within the school environment. As evidence is mixed for the association between student-school connectedness and illicit drug use, with some studies finding a positive association [12] and others finding no association [13], further research on the dimensions of school connectedness and their association with drug use is warranted.

### Limitations and Future Research Directions

Some limitations of the scale are worth noting. For example, as 72% to 92% of the responses fell within the first two response categories, the SPCS scale may be strengthened by expanding the response scale to capture more variance on the social relationship and school connectedness items. As this study was limited to measuring general social support, the scale may be enhanced by further conceptualizing and operationalizing support within the school environment, such as instrumental, motivational, and emotional support [33,34]. Improved operationalization of social support would allow researchers to explore which types of support within the school environment are most important for preventing or reducing selected youth risk behaviors. Distinguishing between the source of social support--be it teacher, classmate, or friend--within the scale may also provide further insights into the social relationships that are most important for protecting against health risk behavior. As this study found that student-teacher connectedness may be a separate factor from student-school connectedness, the scale may be strengthened by including additional measures of the student-teacher relationship, such as fair treatment of students by teachers or personal interest of teachers in how the student is doing [see [29]]. Further research on the validity of the SPSC is also warranted given that our study focused only on two risk behavior outcomes that may be relatively distal to the construct under measurement. Future research on the test-retest reliability of the scale would also provide further understanding of the temporal stability of the measure.

Other limitations that should be considered include the cross-sectional study design for assessing associations with youth risk behavior, which limits our ability to assess causality. Future research is needed to assess both the causal role of school social cohesion in reducing risk behavior in Latin American contexts as well as the mediated pathways between social cohesion and youth risk behavior. Our measures were based on self-report, which may be prone to recall bias as well as social desirability bias in the reporting of risk behaviors and social connectedness. We should also note that the study was limited to those students who were present on the day of the survey. Because the study did not account for truant students, risk behavior prevalence estimates may have been underestimated and the generalizability of the findings to those students is limited. Lastly, further research is needed to expand the tests for reliability and validity of the scale with students from other age groups as well as other Latin American and U.S. Hispanic populations.

## Conclusion

Although a growing body of empirical research, including several adolescent psychosocial theoretical perspectives, points to the importance of the school social environment for influencing both health-promoting and health-debilitating behavior, achieving a more complete understanding of the associations between school social environment and health-risk and health-enhancing behavior among adolescents will require the development of instruments with evidence of strong validity and reliability to measure the school environment. Findings from this study suggest that the Student Perceptions of School Cohesion scale may be a useful instrument for measuring supportive school social relationships and connectedness within a Central American population. The strengths of this scale include: 1) its parsimonious structure based on only 10 items, 2) a 3-factor structure that represents the contextual-level supportive school social climate and individual-level student-school and student-teacher connectedness dimensions of supportive social relationships in school that have been found to be associated with adolescent risk behavior, 3) good internal consistency for the full scale and subscales, and 4) evidence of construct validity based on a large sample of public secondary school students in El Salvador.

## Competing interests

The authors declare that they have no competing interests.

## Authors' contributions

AES and MR conceived of the original study and overall design of the study. AES was responsible for the overall preparation of the manuscript, including the analysis and write-up of the findings and discussion. AM contributed to the drafting and review of the analysis and results section. GQ, MA, and MR provided critical review and revision of the manuscript. All authors read and approved the final manuscript.

## Pre-publication history

The pre-publication history for this paper can be accessed here:

http://www.biomedcentral.com/1472-698X/9/30/prepub
